# Activation of Toll-Like Receptors Differentially Modulates Inflammation in the Human Reproductive Tract: Preliminary Findings

**DOI:** 10.3389/fimmu.2020.01655

**Published:** 2020-08-04

**Authors:** Fahd Benjelloun, Héloïse Quillay, Claude Cannou, Romain Marlin, Yoann Madec, Hervé Fernandez, Fabrice Chrétien, Roger Le Grand, Françoise Barré-Sinoussi, Marie-Thérèse Nugeyre, Elisabeth Menu

**Affiliations:** ^1^MISTIC Team, Virology Department, Institut Pasteur, Paris, France; ^2^Université Paris-Saclay, Inserm U1184, CEA, Immunology of Viral, Auto-immune, Hematological and Bacterial Diseases (ImVA-HB), IDMIT Department, IBFJ, Fontenay-Aux-Roses, France; ^3^Paris Diderot University, Sorbonne Paris Cité, Cellule Pasteur, Paris, France; ^4^Emerging Diseases Epidemiology Unit, Institut Pasteur, Paris, France; ^5^Bicêtre Hospital, AP-HP, Gynecology-Obstetrics Service, Le Kremlin-Bicêtre, France; ^6^Experimental Neuropathology Unit, Institut Pasteur, Paris, France; ^7^International Division, Institut Pasteur, Paris, France

**Keywords:** female reproductive tract (FRT), Toll-like receptors (TLR), cytokines and chemokines, immune cells, mucosa, inflammation

## Abstract

The female reproductive tract (FRT) is the main site of entry of sexually transmitted infections (STIs). Toll-like receptors (TLRs) that recognize pathogenic motifs are widely expressed in the FRT. TLR stimulation induces immune activation and local production of inflammatory mediators. In the FRT, this response should also be compatible with reproductive functions and symbiosis with host microbiota. With a view to develop efficient mucosal vaccines to prevent STI acquisition, the role of TLR ligands in the FRT needs to be explored. We have therefore investigated the cytokine profiles of the different compartments of the FRT (vagina, endocervix, ectocervix, and uterus) before and after stimulation of mononuclear cells from human tissue specimens. The comparison with PBMCs allowed us to highlight the FRT specificities. We first characterized the main immune cell populations in each compartment and observed that their distribution was different through the compartments. The CD45^+^ cells represented a maximum of 11% in the FRT in contrast to 96% in PBMCs. We identified two main populations among the CD45^+^ cells in the four compartments of the FRT: CD3^+^ T cells (CD4^+^ and CD8^+^) and CD14^+^ APCs. B cell populations (CD19^+^) were much less frequent than T cells in all the FRT regions and were equally distributed. NK CD56^+^ cells were detected in all compartments and were more abundant in the uterus. Stimulation of the mononuclear cells was then performed with TLR agonists: R848 for TLR7/8, Poly I:C for TLR3, LPS for TLR4 and ODN CpG for TLR9. Cytokine levels in unstimulated cultures of cells isolated from all FRT compartments were higher than in cultures of unstimulated PBMCs. In contrast, after stimulation with TLR agonists, cytokine responses induced by TLR agonists were moderate in the FRT and significantly lower than in PBMCs. These responses were varied with different TLR ligands and FRT compartments. The cytokine profile induced by TLR activation in the FRT supports the role of these tissues in genital anti-microbial immunity and in the control of inflammation while allowing maintenance of its reproductive function.

## Introduction

The mucosal immune system in the female reproductive tract (FRT) has distinct functions. It provides a protective environment against pathogens, maintains pregnancy, and establishes a well-equilibrated symbiosis between microbiota and host by tolerance mechanisms. The mucosal immune responses in the FRT combine both innate and adaptive immune systems. Epithelial cells and other key immune cells such as macrophages, natural killer (NK) and dendritic cells act against pathogens via sensing through pattern recognition receptors (PRRs) and shape the immune response.

Toll-like receptors (TLRs) are the most studied PRRs in the FRT. These are innate immune receptors, that recognize and sense conserved pathogen associated molecular patterns (PAMPS) produced by potential pathogens but also molecules associated with cell damage (1–3). TLR1, TLR2, TLR4-6, and TLR10 detect microbial ligands at the cell surface, while TLR3 and TLR7-9 recognize pathogen nucleic acid sequences (1, 4, 5). The activation of TLRs induces the production of antiviral and pro-inflammatory cytokines, via the intracellular adaptor protein MyD88 or TRIF, through NFkB or TRIF/TRAM pathways (6–8).

TLRs are widely expressed in the FRT, either in epithelial cells and fibroblasts as well as in immune cells, whose composition varies in the different compartments. TLRs are differently expressed according to the different immune cell populations (9).

Numerous studies highlighted the pivotal role of TLRs in the initiation of the inflammatory reactions that induce immune responses. By influencing the production of cytokines and the maturation of major cell subsets in the FRT (epithelial cells, NK cells, macrophages), TLRs are critically involved in the triggering of adaptive immune responses (5, 10–16).

Understanding the interaction of ligands with their TLRs and their subsequent impact on immune cell populations is primordial for designing new therapeutic molecules that can target TLRs during infectious and inflammatory disease. In fact, different studies showed the critical role of TLRs ligands as adjuvants in vaccines (17–19). In contrast, it has been shown that TLR stimulation in the vaginal mucosa of female macaque induces pro-inflammatory cytokines and type I IFN secretion by pDCs but does not protect them from HIV-1 acquisition (20).

We have previously shown the role of TLR7/8 activation in shaping the macrophages present in the human uterine mucosa (decidua) during pregnancy, by inducing polarization and cytokine secretion. We have also demonstrated the antiviral role of TLR7/8 activation by reducing the susceptibility of decidual macrophages (dM) to HIV-1. TLR7/8 triggering of decidual NK cells induces IFN-γ and TNF-α secretion and influences dM polarization and their permissivity to HIV-1 infection (21, 22).

The aim of this preliminary study was to characterize and to compare the cytokine profiles in the mucosal compartments of the FRT (uterus, endocervix, ectocervix, and vagina) before and after TLR stimulation. In parallel, we also characterized the composition in the main immune cell populations in the samples obtained from the four different compartments.

A better understanding of the properties of the FRT and the modulation of the secretion of soluble factors will help to improve new approaches to strengthen and modulate innate immune responses against pathogens in the different FRT mucosal compartments.

## Materials and Methods

### Tissue Collection, Cell Isolation, and Reagents

FRT samples (endometrium—defined as uterus throughout the manuscript, figures, and tables—, endocervix, ectocervix, and vagina) were obtained from non-menopausal women undergoing surgery for non-malignant etiology at Bicêtre Hospital (Kremlin-Bicêtre, France). All of the women were serologically negative for HIV-1, HBV, and HCV. A summary of the patient population is provided in Tables 1, 2. Surgeries were performed during the proliferative phase of the menstrual cycle. The absence of pathological patterns in tissue samples was confirmed by histopathology analysis. The tissues were minced and digested with 2.5 mg/ml of collagenase IV (Sigma, Saint-Quentin Fallavier, France) and 1 mg/ml of DNase I (Roche, Boulogne-Billancourt, France) for 1 h at 37° C with agitation. The cell suspensions were filtered successively through 100 and 30 μm sterile nylon net cell strainers (BD Biosciences and Miltenyi). Whole mononuclear cells obtained for each FRT compartment were seeded and cultured at 10^6^ cells/ml in F-12 medium and Dulbecco modified Eagle medium (DMEM) containing GlutaMAX (Gibco, Cergy Pontoise, France) (50% vol/vol) supplemented with 15% fetal calf serum (PAA; Les Mureaux, France), penicillin (100 U/ml), and streptomycin (100 μg/ml) and sodium pyruvate 1 mM (Sigma-Aldrich, France).

**Table 1:** Characteristics of the women who participated in the study.

**Women** ***n***	**Samples**	**Average age (Min-Max)**	**Average gravidity (Min-Max)**	**Average parity (Min-Max)**
47	UT (*n* = 28) ENDO (*n* = 21) ECTO (*n* = 24) VAG (*n* = 19)	41.9 years (18–61)	1.6 (0–8)	1.4 (0–4)

**Table 2:** Summary of the main causes of surgeries for the patients.

**Cause of intervention**	**Number of cases**
Fibroma	15
Malformation	8
Bleeding	7
Prolapse	5
Pain	4
Other	8

Peripheral blood mononuclear cells (PBMC) were obtained from the blood of healthy non-menopausal women (Établissement Français du Sang, Saint Louis, France). PBMC were isolated by Ficoll gradient centrifugation using Lymphocyte Separation Medium (Eurobio, Courtaboeuf, France) and cultured at 10^6^ cells/ml in RPMI 1640 medium (Gibco) supplemented with 10% fetal calf serum, penicillin (100 U/ml) and streptomycin (100 μg/ml).

### Flow Cytometry

The cellular composition of the FRT samples (Uterus, *n* = 23; Endocervix, *n* = 17; Ectocervix, *n* = 19; Vagina, *n* = 18, and PBMC, *n* = 6) was analyzed by flow cytometry with at least 2 × 10^5^ cells per sample. Immediately after isolation, cells were stained for 20 min at 4°C with LIVE/DEAD™ Fixable Blue Dead cell stain (Ultra Violet, life technologies, Courtaboeuf, France), anti-CD45 (Amcyan, clone 2D1), anti-CD14 (Pacific Blue, clone M5E2), anti-CD4 (PE-Cy7, clone SK3), anti-CD56 (Alexa700, clone B159), anti-CD19 (FITC, clone HIB19) (Becton Dickinson, Pont de claix, France), anti-CD3 (PE-Texas Red, clone UCHT1), anti-CD8 (APC, clone B9.11) (Beckman Coulter, Villepinte, France). After two washes with PBS/EDTA (2 mM)/SVF (0.5%), cells were fixed in 1% paraformaldehyde. Characterization was performed on an LSRII or a Fortessa (Becton Dickinson Science). The results were analyzed with FlowJo 10.12 software (Tristar).

### Stimulations With TLR Agonists

Stimulations with TLR agonists were performed on whole isolated mononuclear cells obtained from uterus (*n* = 7), endocervix (*n* = 6), ectocervix (*n* = 10), vagina (*n* = 7), and PBMC (*n* = 6). Cells were cultured at 10^6^ cells/ml and stimulated or not for 72 h with TLR agonists (Invivogen, Toulouse, France): R848 at 5 μg/ml for TLR7/8, Poly I:C at 50 μg/ml for TLR3, LPS at 1 μg/ml for TLR4, ODN CpG for class C at 2.5 μM for TLR9. Supernatants were collected after 72 h, cleared (5 min at 2,000 rpm) and stored at −80°C until cytokine and chemokine quantification.

### Cytokine and Chemokine Quantification

Soluble factors in 72 h culture supernatants were quantified by Luminex assay (cytokine human magnetic 25-plex panel; Invitrogen, Courtaboeuf, France). IL-6 and IL-8 were also quantified by ELISA (CXCL8 and Human IL-6 Quantikine ELISA Kit, R&D systems) following the manufacturer's instructions.

### Statistical Analyses

Statistical analyses were carried out with GraphPad Prism software version 7.0. Immune cell populations in the four compartments of the FRT and in PBMCs were compared using the Mann-Whitney test. Lines or bars represent the median. To compare cytokine expression in stimulated vs. non-stimulated condition, fold change was estimated for each donor, and the distribution of the fold changes were tested using a Wilcoxon sign-rank test. The figure legends show in parentheses the number of independent donors used in the experiments. *P* ≤ 0.05 were considered significant.

## Results

### Distinct Immune Cell Distribution in the Human FRT Compartments and PBMC

**Figure 1:**
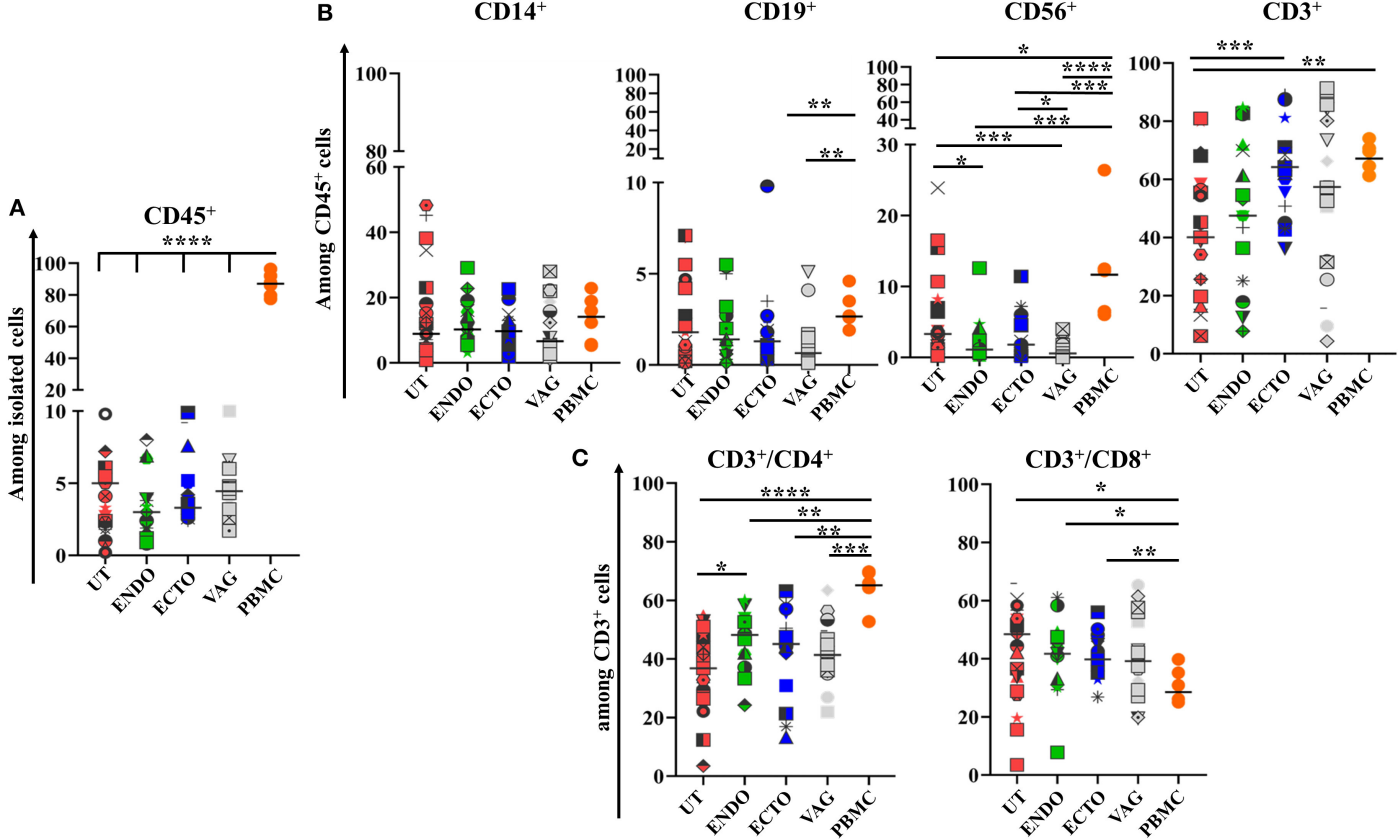
Comparison of the main immune cell populations in the four compartments of the FRT and in PBMCs. **(A)** Percentage of CD45^+^ cells among total isolated cells; **(B)** CD14^+^ Antigen Presenting cells, CD19^+^ B cells, CD56^+^ Natural killer cells, and CD3^+^ T cells among CD45^+^ cells; **(C)** CD3^+^CD4^+^ and CD3^+^CD8^+^ T cells among CD45^+^ cells in the different compartments of the FRT. Uterus (UT) *n* = 23, Endocervix (ENDO) *n* = 17, Ectocervix (ECTO) *n* = 19, Vagina (VAG) *n* = 18, and PBMC *n* = 6. Each symbol represents a sample. The horizontal lines represent the median. Groups are compared by Mann-Whitney test *p < 0.05, **p < 0.01, ***p < 0.005, and ****p < 0.001.

To characterize the main immune cell populations present in the FRT samples studied, the phenotypes of whole isolated mononuclear cells obtained from the different FRT compartments (uterus, endocervix, ectocervix, and vagina) and from PBMC, used as control, were analyzed by flow cytometry. The median proportion of leukocytes (CD45^+^) was 86.7% in the PBMC, 4.7% in the uterus, 3% in the endocervix, 3.3% in the ectocervix, and 4.4% in the vagina (Figure 1A).

**Figure 2:**
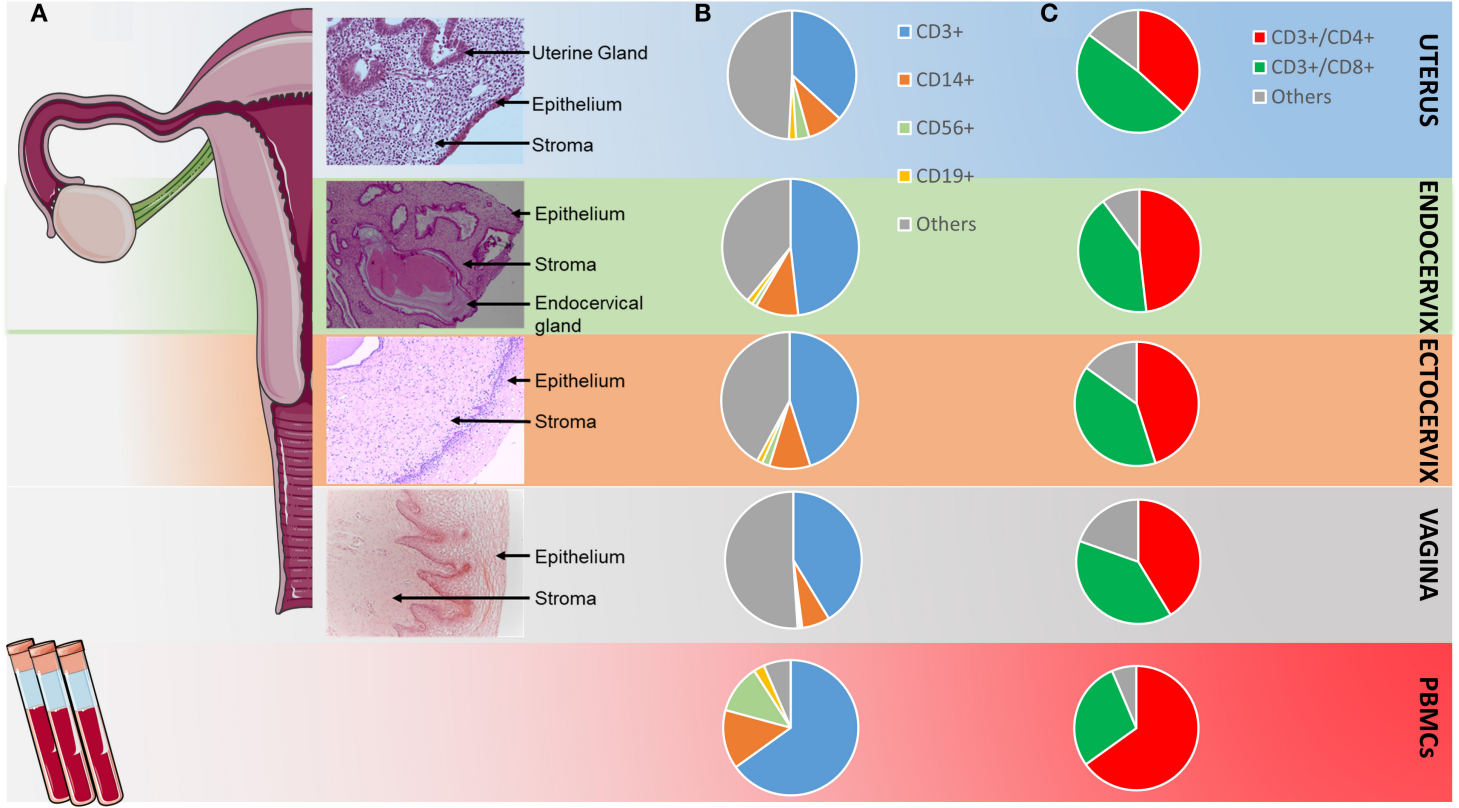
Distribution of the main CD45^+^ cell populations in the FRT and in PBMCs. **(A)** Views of hematoxylin/eosin staining of slides from the four compartments of the FRT; **(B)** repartition of the main CD45^+^ cell populations in the FRT and in PBMC according to flow cytometry analysis shown in Figure 1; “Others” included CD14^−^ mDC, CD123^+^ pDC, neutrophils, ILC3 **(C)** repartition of the CD4^+^ and CD8^+^ cells among the CD45^+^CD3^+^cells in the FRT and in PBMC according to flow cytometry analysis shown in Figure 1. “Others” included CD3^+^CD4^−^CD8^−^ cells.

Among the immune cell populations analyzed, T CD3^+^ lymphocytes were the main leukocyte subpopulation in all the FRT compartments and among the PBMC. They represented 40% of whole leukocytes in the FRT (40.1–67.1%) and more than 60% of the PBMC. They were more abundant in the ectocervix than in the other compartments and statistically greater than in the uterus (*p* < 0.01). The percentage of T CD3^+^ cells was not statistically different in the ectocervix and in the PBMC (Figures 1B, 2). The percentage of CD4^+^ T cells was also statistically higher in the endocervix than in the uterus (48.2 vs. 36.8%, *p* < 0.05). CD4^+^ T cells were statistically more abundant in the PBMC than in the compartments of the FRT. The percentage of CD8^+^ T cells among CD3^+^ were statistically lower in the PBMC (28.5%) than in the compartments of the FRT (48.40% in the uterus, 41.7% in the endocervix, 39.8% in the ectocervix, and 39.1% in the vagina) (Figures 1C, 2). A minor population of CD3^+^ cells was CD4^−^ CD8^−^, amounting to 4.7% of CD3^+^ cells in PBMC, 6.8% in the uterus, 6.5% in the endocervix, 8.8% in the ectocervix, and 6.8% in the vagina (a representative example is shown for PBMC and for the FRT in Supplementary Figure 1). On average, CD14^+^ APCs represented 8.9% in the uterus, 10.2% in the endocervix, 9.7% in the ectocervix, 6.6% in the vagina and 14.1% among PBMC (Figures 1B, 2). The percentages of CD19^+^ B cells were low in the PBMC and in all the compartments of the FRT, they were significantly lower in the ectocervix and in the vagina. They represented 1.8% (median) in the uterus, 1.4% in the endocervix, 1.3% in the ectocervix, 0.6% in the vagina, and 2.6% in the PBMC among the CD45^+^ cells. CD56^+^ NK cells accounted for 3.3% in the uterus, 1.1% in the endocervix, 1.8% in the ectocervix, and 0.5% in the vagina. NK cells were more abundant in the uterus than in the other compartments of the FRT, with a significant difference between the uterus and the ectocervix in comparison with the vagina. NK cells were significantly more abundant in the PBMC (9.4%) than in the FRT (Figures 1B, 2).

Overall, the immune cell populations investigated were present in all mucosal compartments of the FRT and for the PBMC, but with a distinct distribution according to the compartment studied.

### IL-6, IL-8, and CCL2 Were the Most Abundant Cytokines in the FRT

To determine and compare the cytokine profiles in the different compartments of the FRT, the supernatants of unstimulated whole mononuclear cell cultures were collected at 72 h and the cytokines and chemokines were quantified by Luminex or ELISA. Supernatants from unstimulated PBMC were used as control.

**Figure 3:**
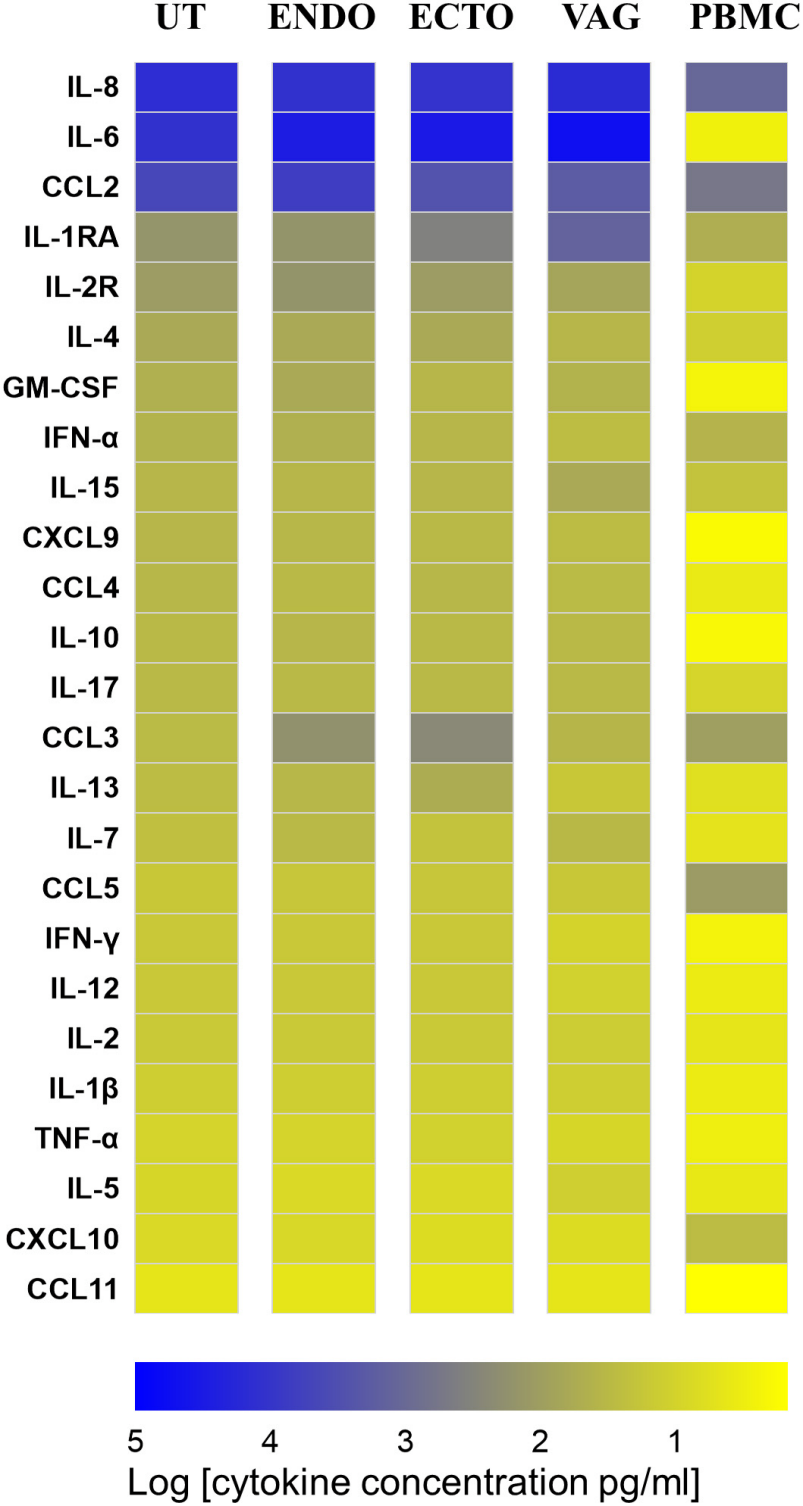
Heat map representing the cytokine and chemokine concentrations in culture supernatants of total isolated cells from the FRT compartments and PBMC in unstimulated condition at 72 h. Uterus (UT) *n* = 7, Endocervix (ENDO) *n* = 6, Ectocervix (ECTO) *n* = 10, Vagina (VAG) *n* = 7, and PBMC *n* = 6. The markers are classified from the most to the less expressed according to the concentrations in the uterus.

IL-6, IL-8, and CCL2 were the most abundant soluble cytokines expressed in all the compartments of the FRT. Except in the uterus, IL-6 was the most abundant in the FRT, with a median of 29,074 pg/ml in the endocervix, 30,822 pg/ml in the ectocervix and 51,812 pg/ml in the vagina, while in the PBMC IL-6 concentration was low in unstimulated conditions (3 pg/ml) (Supplementary Table 1). IL-8 was significantly more concentrated in the vagina than in the endocervix (Supplementary Table 1, Figure 3, *p* ≤ 0.05). IL-8 and CCL2 were significantly more abundant in the compartments of the FRT than in the PBMC (Supplementary Table 1, Figure 3). Interestingly, anti-inflammatory markers such as IL1-RA, IFN-α, and CCL3 were distinctly expressed in the FRT. IL1-RA was significantly more abundant in the vagina and in the ectocervix than in the uterus and more abundant in all compartments of the FRT compared to PBMC. While type I IFN-α was significantly more concentrated in the endocervix than in other FRT compartments. CCL3 was higher in the cervix and significantly higher between the ectocervix and the uterus (Supplementary Table 1, Figure 3).

CCL5 was significantly less abundant in the FRT than in the PBMC as well as CXCL10 in the ectocervix and in the vagina compared to the PBMC while IL-2R, IL-4, GM-CSF, IL-15, CCL4, IL-10, IL-17, IL-7, IL-12, IL-1β were significantly more concentrated in all the compartments of the FRT than in the PBMC.

In all the FRT compartments as well as in the PBMC, IL-13, IL-17, IFN-γ, IL-2, TNF-α, IL-5, and CCL11 were very weakly expressed, median values were close to the thresholds of detection.

### In the FRT, TLR Stimulation Modulated Cytokine Production Mainly in the Cervix

To decipher the impact of TLR stimulation of isolated mononuclear cells of the different FRT compartments, we compared the cytokine profiles in the 72 h culture supernatants of unstimulated and TLR stimulated cells (TLR3 with poly I:C, TLR4 with LPS, TLR7/8 with R848 and TLR9 with ODN CpG). PBMC were used as controls in the same conditions.

**Figure 4:**
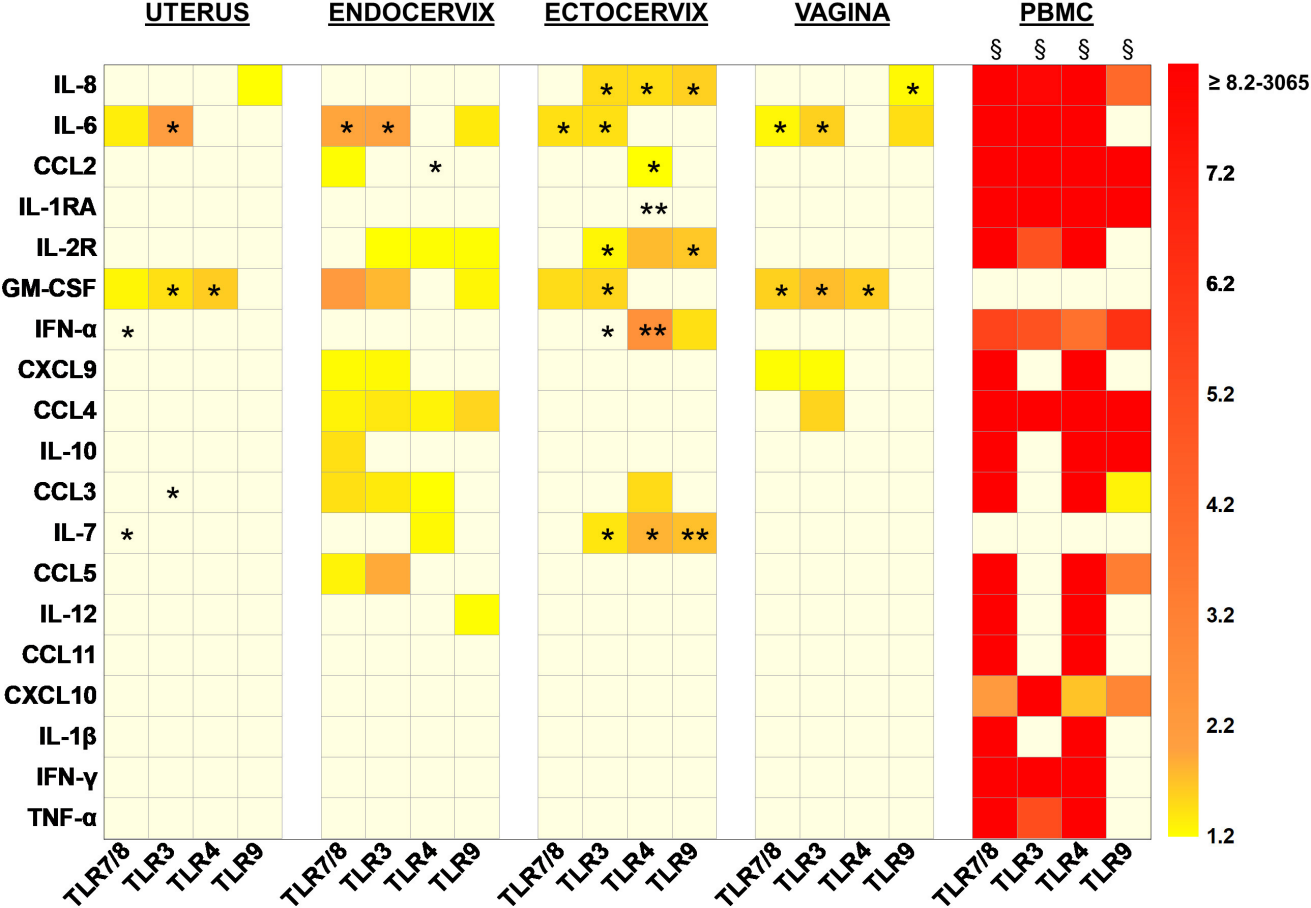
Heat map representing the increased fold changes of cytokine concentration in culture supernatants of total isolated cells from the FRT compartments and PBMC stimulated with TLR7/8, TLR3, TLR4, and TLR9 agonists at 72 h. Uterus *n* = 7, Endocervix *n* = 6, Ectocervix *n* = 10, Vagina *n* = 7, and PBMC *n* = 6. A Wilcoxon single-rank test was performed: *p < 0.05, **p < 0.01, and ^§^*p* < 0.0001. Each case represents the median of fold change for one compartment for one cytokine. Only increased fold changes ≥1.2 are represented.

The cytokines and chemokines were quantified by Luminex or ELISA. The concentration of each molecule in each condition has been reported to the unstimulated condition as described above, and these results were used to calculate fold changes (Figure 4).

When TLR7/8 were stimulated, pro-inflammatory markers such as IL-6 and GM-CSF were increased in all the compartments. In the endocervix the over expression of IL-6 and GM-CSF was accompanied with an increase of chemokines and cytokines with a dominance of lymphocyte attractant chemokines such as CCL2, CXCL9, CCL3, CCL4, CCL5, and IL-10.

The activation of TLR3 also induced an increased production of IL-6 and GM-CSF in all of the compartments. In the cervix (endo and ecto) the impact of the stimulation of TLR3 on cytokine production increase was broader. More specifically, IL-2R, CXCL9, and CCL3/4/5 levels were increased in the endocervix and significantly IL-8, IL-2R, IFN-α, and IL-7 in the ectocervix.

The triggering of the TLR4 induced a significant overexpression of GM-CSF in both the uterus and vagina. The impact was more important in the cervix since CCL2, IL-2R, CCL3, and IL-7 were increased after the activation of TLR4. Furthermore, IL-8, IL-1RA, IFN-α were overexpressed in the ectocervix and CCL4 in the endocervix.

When TLR9 was activated, IL-8 secretion was enhanced in all the compartments of the FRT except the endocervix. In the vagina and in the endocervix the production of IL-6 was increased. The overexpression of IL-2R, GM-CSF, CCL4, and IL-12 was also observed in the endocervix. TLR9 activation induced an increased secretion of IL-2R, IFN-α, and IL-7 in the ectocervix (Figure 4).

In the FRT, the production of CCL11, CXCL10, IL-1β, IFN–γ, and TNF-α was never increased whatever the agonist used for the stimulation.

Furthermore, none of the TLR stimulations modified the production of CCL2, IL-1RA, IL2R, CXCL9, CCL4, IL-10, CCL5, and IL-12 in the uterus; the production of IL-8, IL-1RA, and IFN-α in the endocervix; the production of CXCL9, CCL4, IL-10, CCL5, and IL-12 in the ectocervix; and the production of CCL2, IL-1RA, IL-2R, IFN-α, IL-10, CCL-3, IL-7, CCL5, and IL-12 in the vagina. The cervix and in particular the ectocervix, were the FRT compartments the most susceptible to TLR activation since the production of cytokines was significantly activated in these compartments.

In PBMC, the stimulation of the TLRs, induced a broad and strong increase production for all the markers studied except GM-CSF and IL-7. PBMC were more responsive to TLR7/8 and TLR4 stimulations which increased production of the same cytokines. TLR3 and TLR9 stimulations induced distinct cytokines in the PBMC: TLR3 induced IL-6, IL-2R, IFN-γ, and TNF-α while TLR9 induced IL-10, CCL3, CCL5, both stimulations increased IL-8, CCL2, IL-1RA, IFN-α, CCL4, CXCL10.

The production of IL-4, IL-15, IL-17, IL-13, IL-2, and IL-5 never increased in the FRT nor the PBMC whatever the TLR stimulation used (data not shown).

## Discussion

The FRT mucosa is the principal portal of entry of STIs, and the TLRs are the main receptors of innate immunity. The activation of TLR initiates and guides immune responses. Different determinants shape these responses, among them the composition of immune cells in a specific compartment or the presence of pre-existing inflammation. Better understanding of the mechanisms of protection of the FRT mucosae and of the factors involved will give insights for new approaches with which to modulate immunity in the different FRT mucosae. In this study we aimed to decipher the impact of TLR activation (TLR3, TLR4, TLR7/8, and TLR9) on the profile of secretion of soluble factors such as cytokines and chemokines in the mucosal environment and to compare these profiles in the different compartments of the FRT.

In the first part of this study, we characterized the main immune cell populations in the human FRT and confirmed that immune cells are compartmentalized. Immune cells in the studied samples represented about 11% maximum of the isolated cells, which is coherent to what has been previously described (23, 24). We identified two main populations among the CD45^+^ leukocytes in the four compartments of the FRT: CD3^+^ T cells (CD4^+^ and CD8^+^) and CD14^+^ APCs. T cells were more abundant in the cervix than in the uterus, while the APCs were similarly abundant in all compartments of the FRT. The median proportions of CD3^+^CD8^+^ T cells were higher in all FRT compartments compared to PBMCs. CD3^+^CD4^−^CD8^−^ cells were detected in all compartments and may include γδ1 T cells which have been shown to play an important role in mucosal integrity, protection against pathogens and in reproduction (25). B cell populations (CD19^+^) were much less frequent than T cells in all mucosae of the FRT and were equally distributed. The NK CD56^+^ cells were more abundant in the uterus and the ectocervix than in the vagina. These results are in agreement with those described in previous studies (24, 26). Indeed, similar distribution of immune cells in the FRT have been reported, it has been described that B cells and monocytes are present in all the compartments of the FRT and that both CD4^+^ and CD8^+^ T lymphocytes are major immune cell populations in all the mucosae of the FRT. In addition, Wira et al., have observed that aggregates of B cells, surrounded by T cells and macrophages were present in the upper tract of the FRT and that in the lower tract, DC, macrophages and lymphocytes were present. NK cells were detected in all the compartments of the FRT (26–29).

A large variability in the percentage of immune cell subpopulations has been observed within a same compartment in our study. No correlation was found between cell composition and cause of surgical intervention (fibroma and other etiologies such as malformation, bleeding, prolapse, pain…). Additional clinical factors, including hormonal status, inflammation, and/or sexual activity might influence immune cell composition in the reproductive tract. All the tissues of this study were obtained during the proliferative phase of the menstrual cycle when the estrogen levels are high and progesterone levels are low.

This study showed that each mucosal compartment of the FRT has its own immune cell composition together with some common features and a high intra-individual variability.

It would be informative to perform an extensive identification of the subpopulations present in the different compartments of the FRT to identify more specific cells amongst the T cells, NK, or APC such as memory T cells, Tregs, mDC, and pDC or ILC (Innate lymphoid Cell) populations but the number of cells obtained in humans are often limiting to perform such a detailed characterization. We have previously published a more detailed characterization of immune cell populations present in the different compartments of the FRT in cynomolgus macaques (30).

Furthermore, given the fact that local epithelial and endothelial cells produce several inflammatory cytokines, it is tempting to speculate that resident non-lymphoid cells may also influence TLR responsiveness in the different regions of the FRT, an issue which will need to be addressed in future studies.

In the second part of this study, we measured the cytokines and chemokines produced by the isolated mononuclear cells of each compartment of the FRT, either spontaneously or after stimulation with TLR agonists in 72 h culture supernatants. The comparison with PBMCs allowed us to highlight the specificities of the FRT mucosae. In previous studies, stimulation of TLR has been performed on primary or epithelial cell lines (31–35) or on cervical mononuclear cells (36). In this study we have performed the stimulation on the whole mononuclear cell populations isolated from the different mucosae of the FRT. The soluble factor concentrations measured were as such the result of the production by the combination of the different cell populations and not only by the epithelial cells. This is important because immune cells express TLR and react to TLR stimulation. Furthermore, we were able to study the four compartments of the FRT in the upper and lower tracts and to compare them.

Chemotactic (CCL2, IL-8) and pro-inflammatory (IL-6) cytokines were the most abundant soluble markers in all the compartments of the FRT in the unstimulated conditions. IL1-RA and IL-2R were also broadly expressed in the FRT. GM-CSF, IFN-α, CCL4, IL-10, CCL3, CCL5, and IL-1β were significantly detected in some samples. IL-1RA is significantly more abundant in the vagina than in the ectocervix and the uterus. The β-chemokine CCL3 is present in all the compartments of the FRT but is significantly more abundant in the ectocervix than in other compartments. In PBMCs, CCL2, IL-8, and CCL5 are the most abundant cytokines produced in unstimulated conditions. For all the cytokines (except for CCL5), the basal expression levels were higher in the FRT than in PBMCs. This could be explained by the fact that the reproductive tract, especially the lower tract, is constantly exposed to commensal and pathogenic microorganisms which induce TLR signaling and cytokine production. The upper tract is considered to be sterile and the immune system should inhibit the dissemination of microorganisms in this compartment that would otherwise be detrimental for reproductive functions (16, 36). It has been shown that the uterus can be contaminated with bacteria upon ascending migration during sexual intercourse (37).

In contrast, after stimulation of the TLRs (TLR 3, 4, 7/8, and 9), PBMCs were more reactive than mononuclear cells from the FRT with an activation of the production of several soluble factors with much higher fold increases than in the FRT. On the contrary, two cytokines, GM-CSF and IL-7, which are activated in the FRT after TLR stimulation, were not stimulated in PBMCs. In PBMCs, 9–17 cytokines were enhanced with fold changes going up by more than 3 logs (3,065 for CCL4 with TLR7/8 stimulation). The most efficient stimulation in PBMCs was via TLR7/8, then TLR4, TLR3, and the less efficient was via TLR9. In the FRT, significant over-expressions were mostly observed in the cervix with 2–8 cytokines enhanced with a maximum fold change of 2.5 (IFN-α in the ectocervix with TLR4 stimulation). Each TLR stimulation induced a specific cytokine profile according to the FRT compartment studied.

These data suggest that despite the ability of the mononuclear cells of the FRT to respond to TLR stimuli, the response is controlled and moderate compared to the response in PBMCs 72 h after stimulation. The 72 h kinetics were chosen according to our previous paper reporting TLR stimulation of human decidual cells (38). At 72 h all cytokines were detected which was not the case with earlier time points depending on the type of TLR stimulated. For some of the cytokines tested, the concentration was lower at 72 h than at 24 h. A full kinetics study in the PBMC is presented in Supplementary Figure 2.

An increase of TLR expression in the FRT has been reported in the secretory phase of the menstrual cycle (39), it will be interesting to investigate whether the response to stimuli would be different. We should mention that circulating cells are also influenced by menstruation (40) but in contrast to the FRT tissue donors, it was not possible to know the menstrual cycle of donors at the time of sampling.

If TLR ligands alone do not induce a high cytokine response, TLR ligands together with short chain fatty acids, which are produced at high levels by anaerobic bacteria in bacterial vaginosis, have been shown to increase a high production of pro-inflammatory cytokines (41) that might explain a higher susceptibility to STIs.

It will be of particular interest to evaluate the influence of the microbiota composition and associated metabolites in the modulation of TLR responsiveness in the different FRT tissue compartments, an issue which we plan to address in a separate study.

In a murine model, studies using TLR ligands delivered intravaginally have shown complete protection against HSV2 but the results are more contrasted concerning HIV-1 (42).

TLR agonists are often used as adjuvants to improve the immune responses to vaccines mostly through the parenteral route. With a view to develop future mucosal vaccines to prevent STIs, it is necessary to understand the impact of TLR ligands locally. Our study gives some understanding on this aspect.

In conclusion, we report here that FRT mucosae present a distinct composition in immune cell populations and cytokine profiles. Each TLR stimulation induces a moderate enhancement of a specific cytokine profile production characteristic of each compartment. The cytokine profiles induced by TLR activation in the FRT are consistent with the initiation of antiviral or antimicrobial responses together with the control of inflammation compatible with the reproductive functions.

## Data Availability Statement

The raw data supporting the conclusions of this article will be made available by the authors, without undue reservation.

## Ethics Statement

The studies involving human participants were reviewed and approved by for tissue samples: the Comité de Protection des Personnes (n° 2014/42NICB), the Assistance Publique des Hôpitaux de Paris (no. VAL/2016/2011.277/01), the clinical research committee of the Institut Pasteur, Paris, France (no. 2013.23), and the Comité National de l'Informatique et des Libertés (no. 911472 v3). For blood donors: Établissement Français du Sang, Saint Louis, France; C CPSL UNT-N°13/EFS/101. The patients/participants provided their written informed consent to participate in this study.

## Author Contributions

FB, HQ, FB-S, M-TN, and EM: study conception and design. HF: clinical supervision and sample collection. FB, HQ, CC, and M-TN: acquisition of data. FB, YM, FC, M-TN, and EM: analysis and interpretation of data. FB and EM: drafting of manuscript. YM, RM, RL, FB-S, M-TN, and EM: critical revisions. All authors contributed to the article and approved the submitted version.

## Conflict of Interest

The authors declare that the research was conducted in the absence of any commercial or financial relationships that could be construed as a potential conflict of interest.
