# Supplementary material for: Activation of Toll-Like Receptors Differentially Modulates Inflammation in the Human Reproductive Tract: Preliminary Findings
Source: Front Immunol. 2020 Aug 4;11:1655. doi: 10.3389/fimmu.2020.01655 (PMC7417306; doi:10.3389/fimmu.2020.01655)
Supplement: Supplementary file 1 [file Data_Sheet_1.PDF]

Activation of Toll-Like Receptors differentially modulates inflammation in the human reproductive tract : Preliminary findings

Fahd Benjelloun[1,2], Héloïse Quillay[1,3], Claude Cannou[1,2], Romain Marlin[2], Yoann Madec[4], Hervé Fernandez[5], Fabrice Chrétien[6], Roger Le Grand[2], Françoise Barré-Sinoussi[7], Marie-Thérèse Nugeyre[1,2], Elisabeth Menu[1,2].

[1]MISTIC Team, Virology departement, Institut Pasteur, Paris, France;
[2]Université Paris-Saclay, Inserm U1184, CEA, « Immunology of Viral, Auto-immune, Hematological and Bacterial Diseases (ImVA-HB), IDMIT Department, IBFJ, Fontenay-Aux-Roses, France;
[3]Paris Diderot University, Sorbonne Paris Cité, Cellule Pasteur, Paris, France;
[4]Epidemiology of emerging diseases Unit, Institut Pasteur, Paris, France;
[5]Bicêtre hospital, AP-HP, Gynecology-Obstetrics service, Kremlin Bicêtre, France ;
[6]Experimental neuropathology Unit, Institut Pasteur, Paris, France;
[7]International division, Institut Pasteur, Paris, France.

Supplementary table 1

	Uterus Median (Min-Max) n=7	Endocervix Median (Min-Max) n=6	Ectocervix Median (Min-Max) n=10	Vagina Median (Min-Max) n=7	PBMC Median (Min-Max) n=6	p values			
						Ut	Endo	Ecto	Vag
**IL-8**	13774 (7667-16941)	11629[#] (7955-13587)	10317 (6264-17332)	15019[#] (9748-18893)	1067 (307-6184)	*	*	*	*
**IL-6**	11315 (847-81329)	29074 (4173-66554)	30822 (3050-204170)	51812 (8757-440730)	3 (3-170)	*	*	*	*
**CCL2**	4394 (608-13842)	6533 (202-10665)	2626 (642-14374)	1751 (180-15320)	527 (7-1665)	*	*	*	-
**IL-1RA**	145 [$§] (145-635)	153 (145-5467)	346[$] (145-3947)	1281[§] (327-6067)	49 (49-1121)	*	*	*	*
**IL-2R**	106 (29-225)	152 (64-202)	108 (43-185)	73 (36-179)	10 (10-88)	*	*	*	*
**IL-4**	58 (35-58)	58 (35-58)	58 (35-58)	35 (35-58)	12 (12-12)	*	*	*	*
**GM-CSF**	44 (7-1206)	57 (24-224)	34 (7-217)	41 (24-128)	2 (2-2)	*	*	*	*
**IFN-α**	38 (23-89)	45[§#] (31-103)	35[#] (11-39)	25[§] (11-102)	38 (4-53)	-	-	-	-
**IL-15**	35 (35-59)	35 (35-59)	35 (35-59)	59 (35-59)	20 (20-20)	*	*	*	*
**CXCL9**	34 (16-84)	32 (16-39)	31 (12-45)	28 (16-48)	2 (2-89)	*	-	*	*
**CCL4**	33 (11-215)	30 (20-181)	33 (11-214)	29 (11-773)	4 (4-140)	*	*	*	*
**IL-10**	30 (5-50)	34 (11-275)	30 (9-131)	30 (5-305)	2 (2-23)	*	*	*	*
**IL-17**	30 (27-30)	30 (27-30)	30 (27-30)	30 (27-44)	9 (9-9)	*	*	*	*
**CCL3**	29[#] (25-704)	176 (25-1319)	245[#] (26-1053)	35 (25-2 322)	94 (8-207)	*	-	*	-
**IL-13**	26 (16-85)	33 (16-67)	53 (16-74)	16 (16-40)	5 (5-57)	-	-	*	-
**IL-7**	23 (14-42)	31 (20-39)	20 (2-35)	31 (17-39)	5 (5-5)	*	*	*	*
**CCL5**	16 (16-36)	17 (16-118)	17 (16-82)	16 (16-37)	113 (53-381)	*	*	*	*
**IFN-γ**	16 (7-16)	16 (8-16)	16 (7-16)	10 (8-16)	2 (2-35)	-	-	-	-
**IL-12**	15 (10-19)	15 (10-18)	15 (10-18)	10 (10-16)	3 (3-3)	*	*	*	*
**IL-2**	15 (13-15)	15 (13-15)	15 (13-15)	13 (13-15)	4 (4-4)	*	*	*	*
**IL-1β**	12 (10-283)	12 (10-108)	12 (10-59)	12 (10-65)	3 (3-3)	*	*	*	*
**TNF-α**	9 (9-43)	9 (9-12)	11 (9-14)	9 (9-12)	3 (3-34)	*	*	*	*
**IL-5**	8 (7-11)	7 (7-11)	7 (7-11)	11 (7-11)	4 (4-65)	-	-	-	-
**CXCL10**	7 (6-117)	8 (6-36)	6 (6-34)	6 (6-7)	28 (2-38)	-	-	*	*
**CCL11**	4 (4-4)	4 (4-4)	4 (4-5)	4 (4-4)	1 (1-20)	-	-	-	-

Supplementary Table 1: Cytokine and chemokine concentrations in 72h-supernatants of total isolated cells from the FRT compartments and PBMC in unstimulated condition. Right panel represents the PBMC profiles and the statistical difference with each compartment of the FRT. Uterus (Ut) n=7, Endocervix (Endo) n=6, Ectocervix (Ecto) n=10, Vagina (Vag) n=7 and PBMC n=6. The markers are classified from the most to the less expressed according to the concentration in the uterus. Each case corresponds to the median with the min and max concentration measured. The compartments are compared with a Mann-Whitney test. Symbols (#§$*) represent a statistically significant difference of median values between two different compartments with a p value ≤0.05. Symbol (-) represents no statistical difference between two compartments.

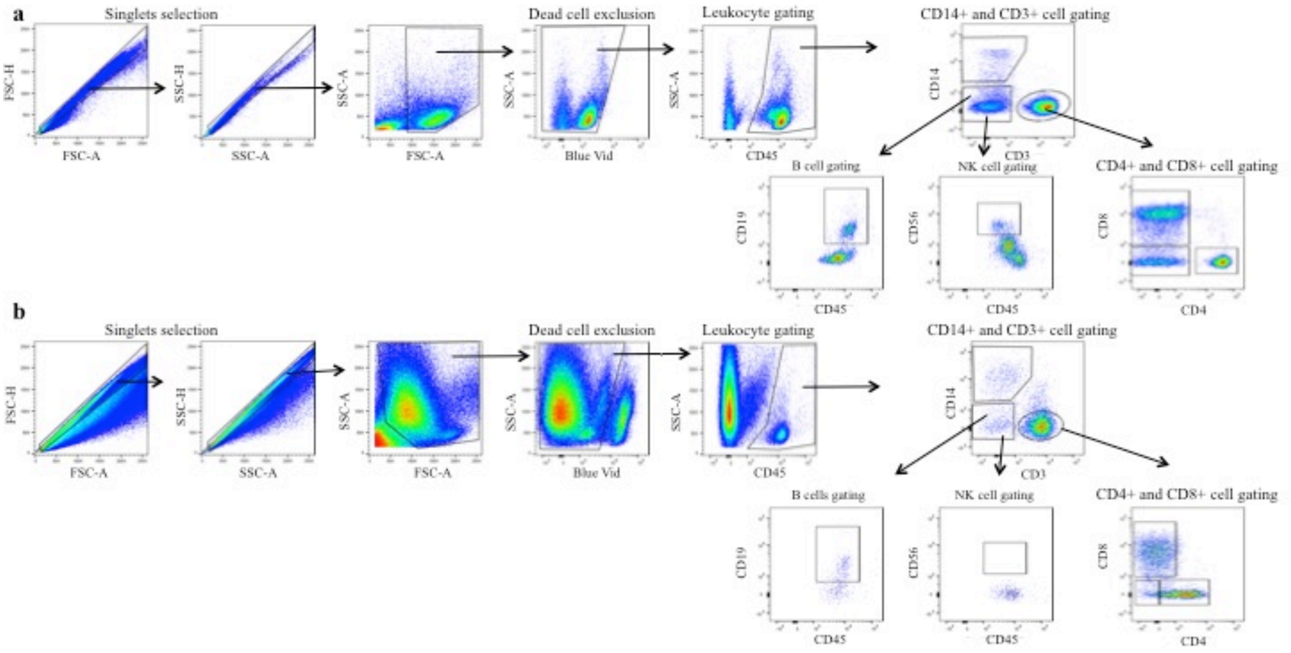

Supplementary Figure 1. Gating strategy used to determine the different cell populations.

The same gating strategy was used for PBMC (a) and for the different compartments of the FRT, one example of ectocervix is shown in (b).

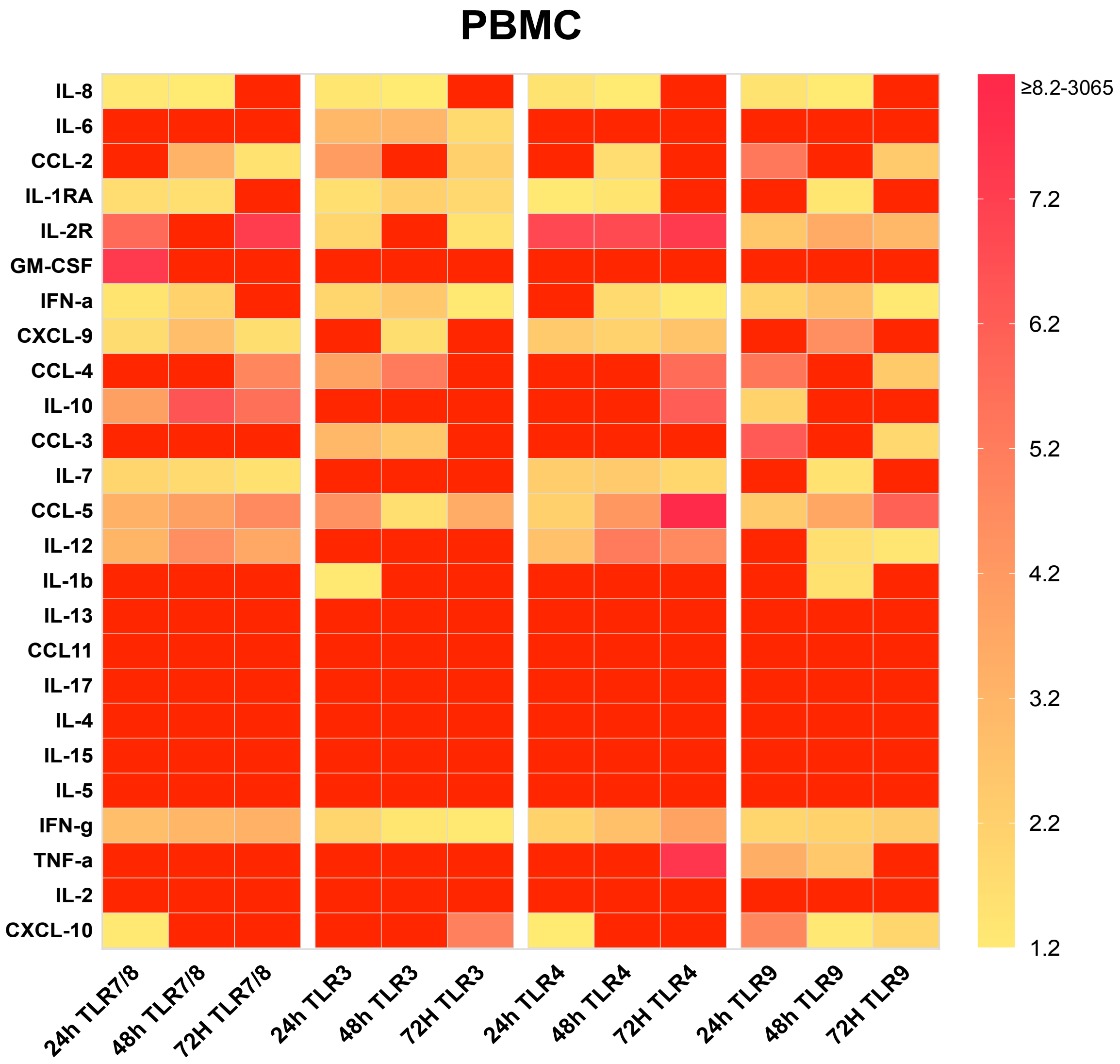

Supplementary Figure 2. Heat map representing the increased fold changes of cytokine concentration in culture supernatants of PBMC stimulated with TLR7/8, TLR3, TLR4 and TLR9 agonists at 24h, 48h and 72h. Each case represents the median (n=6) of fold change for one cytokine. Only increased fold changes ≥1.2 are represented.
